# Comparative Efficacy and Safety of Sildenafil, Tadalafil, Vardenafil, Mirodenafil, Coenzyme Q, and Testosterone in the Treatment of Male Sexual Dysfunction: A Systematic Review and Meta-Analysis

**DOI:** 10.1192/bjo.2025.10790

**Published:** 2025-06-20

**Authors:** Gaurav Uppal, Ashok Shenoy, S Sayan, Betsy Marina Babu, Asha Devi Dhandapani

**Affiliations:** 1 Satyam Hospital, Ludhiana, India; 2 Kasturba Medical College, Mangalore, India; 3 Manipal University, Jaipur, India; 4 London and KSS School of Psychiatry, London, United Kingdom; 5 BCUHB, Wrexham, United Kingdom

## Abstract

**Aims:** This study through systematic review approaches and meta-analysis aimed to determine safe drug options for treating male sexual dysfunction due to erectile dysfunction by comparing sildenafil, tadalafil, vardenafil, mirodenafil, coenzyme Q and testosterone.

**Methods:** Systematic review methodology was used to retrieve data from complete database searches done in PubMed/MEDLINE, Embase, and Cochrane Central Register of Controlled Trials. Randomized controlled trials research designs with adult male participants who presented with erectile dysfunction on sildenafil, tadalafil, vardenafil, mirodenafil, coenzyme Q and testosterone were included as part of the review. The Cochrane Risk of Bias version 2.0 was utilized by two independent reviewers for assessing bias risk and extracting data. Sensitivity analysis was used to reduce the risk of bias.

**Results:** Phosphodiesterase inhibitors type 5 (PDE 5) are the most effective medications for erectile dysfunction (ED). Participants on sildenafil had effective erections between 77–84% at doses of 50–100 mg and tadalafil emerged as the “Weekend pill” because of its 36-hour maximum effect duration of action. The concentration-focused activity along with higher potency values of vardenafil make it superior to other PDE5 inhibitors. The newly developed therapeutic medication mirodenafil exhibits better PDE5 selectivity than existing drugs within its operational mechanism. Minor variations were noted as a cohort of participants preferred tadalafil above sildenafil. Testosterone supplementation is beneficial for when monotherapy with a PDE5 inhibitor has not been effective.

**Conclusion:** Being the first-line treatment for erectile dysfunction, PDE5 inhibitors should be prescribed based on the distinctive pharmacological attributes they possess. Medical treatment selection methods must take into account both the need of patients as well as their preference. The recent introduction of mirodenafil medication has opened new possibilities in the medical treatment of ED. The effectiveness of ED treatment brings optimistic outcomes to the quality of life to hundreds of millions of men across the globe and further studies will drive medical progress to improved treatment modalities including stem cell therapy.

